# Pituitary metastasis: an unexpected complication in advanced cancer

**DOI:** 10.11604/pamj.2025.50.41.46622

**Published:** 2025-02-04

**Authors:** Natthaphon Wonghakaeo, Thiti Snabboon

**Affiliations:** 1 Department of Internal Medicine, Suddhavej Hospital, Faculty of Medicine, Mahasarakham University, Maha Sarakham Province, Thailand,; 2 Department of Medicine, Faculty of Medicine, Chulalongkorn University, Bangkok, Thailand,; 3 Excellence Center in Diabetes, Hormone, and Metabolism, King Chulalongkorn Memorial Hospital, Thai Red Cross Society, Bangkok, Thailand

**Keywords:** Hypopituitarism, diabetes insipidus, breast cancer, pituitary metastasis

## Image in medicine

A 57-year-old man with metastatic breast cancer presented with a four-month history of polyuria, which was diagnosed as diabetes insipidus (DI). Further hormonal evaluation revealed concurrent hypothyroidism and hypocortisolism. Magnetic resonance imaging demonstrated a pituitary lesion with an absent posterior bright spot, suggesting a metastatic lesion. Unfortunately, the patient died from uncontrolled advanced disease one month later. Pituitary metastasis is rare but increasingly recognized due to advances in imaging and treatment. Breast and lung cancers are the most common primary malignancies. Clinical presentations vary from asymptomatic cases to visual field defects or pituitary dysfunction caused by compression of adjacent structures. Diabetes insipidus is the most common endocrine abnormality, with anterior hypopituitarism in 25-45% of cases. Diagnosing pituitary metastasis can be challenging, as it may be mistaken for pituitary adenoma. Key magnetic resonance imaging (MRI) findings indicative of metastasis include dumbbell-shaped lesions, stalk thickening, erosion of the sella turcica, and the absence of the posterior pituitary bright spot. The prognosis is generally poor, with a median survival of 12.9 months, and no treatment has significantly improved survival. Therefore, clinicians should consider pituitary metastasis, especially in patients with advanced malignancies presenting with DI.

**Figure 1:**
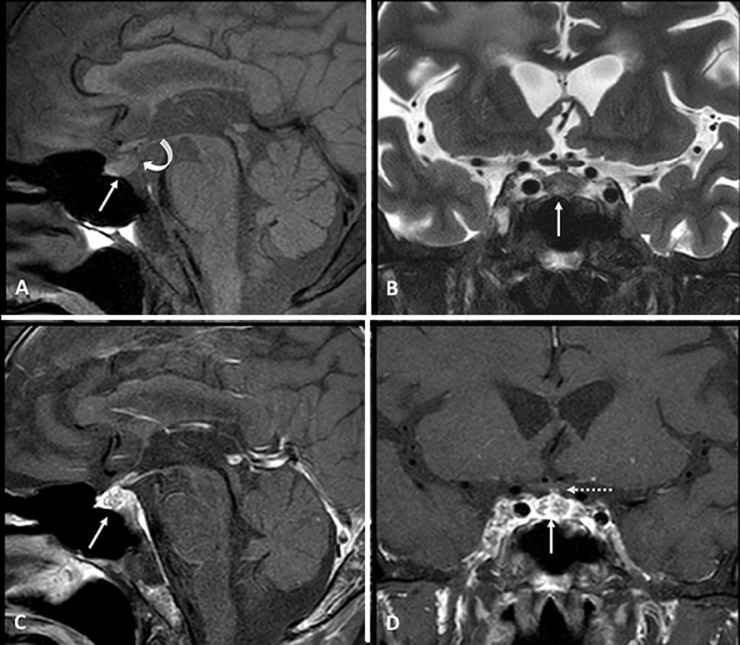
magnetic resonance imaging findings; A) sagittal T1-weighted image showing a 1 cm hypointense lesion in the mid-posterior part of the pituitary gland (arrow), with an absent posterior bright spot (curved arrow); B) coronal T2-weighted image demonstrating a hyperintense lesion (arrow); C, D) post-gadolinium sagittal and coronal T1-weighted images revealed a heterogeneous enhancement lesion (arrow) with a normal-appearing pituitary stalk (dashed arrow); metastatic lesions were also noted in the clivus and occipital bone

